# Implementation, integration, and institutionalization of a community-based strategy for hypertension control in Colombia: the RE-HOPE implementation study protocol

**DOI:** 10.3389/fcvm.2026.1790340

**Published:** 2026-04-15

**Authors:** Jose P. Lopez-Lopez, Johanna Otero, Alvaro Castañeda-Hernández, Daniel Martinez-Bello, Claudia Garcia, Claudia Torres, Yessica Giraldo-Castrillon, Yeny Castellanos-Dominguez, Yuri Sanchez-Martinez, Nathalia Barbosa, Patricio López-Jaramillo

**Affiliations:** 1 Masira Research Institute, Universidad de Santander (UDES), Bucaramanga, Colombia; 2 Universidad de Santo Tomas—Seccional Bucaramanga, Bucaramanga, Colombia

**Keywords:** cardiovascular risk factors, community health workers, hypertension, implementation science, primary health care

## Abstract

**Background:**

Hypertension is the leading modifiable risk factor for cardiovascular disease, particularly in low- and middle-income countries (LMICs). Despite the effectiveness of pharmacological and non-pharmacological interventions, control rates remain low. Therefore, novel community-based strategies are needed for enhancing the detection, treatment, and control of hypertension. Our aim will be to implement and evaluate the results of a community-based strategy to reduce blood pressure (BP) levels in a LMIC.

**Methods:**

We describe the “Implementation, Integration, and Institutionalization of a Community-Based Care Program to Reduce Cardiovascular Risk in Santander” (RE-HOPE) study protocol. Adults aged ≥18 years, with *de novo* or uncontrolled hypertension, or with hypertension not participating in cardiovascular risk management programs, will be included. Individuals will be identified through active community screening. The intervention will consist of early identification of hypertension, enrolling participants in primary care institutions, and conducting follow-up home visits. These visits will include counseling on medication adherence and lifestyle modifications, as well as training for healthcare providers and community health workers (CHWs). Referrals to primary care providers and follow-up appointments will be coordinated by CHWs. The primary outcome will be change in BP levels from baseline to 12 months of follow-up.

**Results:**

We expect to generate evidence about implementation outcomes, such as feasibility and acceptability of community-based screening, the adoption of this approach by primary care centers and fidelity of activities developed by CHWs. In addition, the implementation of this model is anticipated to reduce the prevalence of uncontrolled hypertension and minimize barriers to healthcare access. It is also expected that this strategy facilitates the timely detection and monitoring, address institutionalization gaps, and ultimately reduce the long-term burden of hypertension.

**Conclusions:**

The RE-HOPE study will provide evidence on the effectiveness and implementation of a community-based hypertension control strategy integrated into primary care services. This strategy may serve as a replicable approach for improving hypertension control in low- and middle-income countries (LMICs).

## Introduction

Cardiovascular diseases are the leading cause of morbidity and mortality worldwide, with hypertension being the risk factor accounting for the highest population-attributable risk fraction (1, 2). Despite the availability of effective pharmacological treatments and non-pharmacological interventions, hypertension control remains suboptimal, contributing significantly to the global burden of disease and disability-adjusted life years (DALYs) (3). In the Americas, hypertension prevalence ranges from 18% to 60%, exceeding 40% in Caribbean countries. However, control rates remain low, 35% among women and 23% among men (4) In Colombia, the May Measurement Month 2019 campaign reported that 27.9% of participants had hypertension, 60% of whom were receiving treatment, yet only 38.4% had adequate blood pressure control (5). These figures have prompted the emergence of innovative strategies at both the individual and population levels to improve hypertension detection, management, and control, particularly in low- and middle-income countries (LMICs). Novel approaches include multidisciplinary care models involving non-physician health workers, continuous training of healthcare staff, task shifting, virtual care/telemedicine, the use of simplified diagnostic algorithms, and pharmacologic optimization through fixed-dose combination therapy (2, 6, 7). Indeed, multicomponent strategies that incorporate task-shifting and non-physician-led medication administration into standard care have demonstrated effectiveness in reducing systolic blood pressure by an average of 6.6 mmHg (95% CI: −9.0 to −4.2) (8). Community-based care models have also proven effective in managing hypertension in populations with limited healthcare access (9). Task-shifting interventions that engage community health workers (CHWs) enable them to perform key functions, such as screening, risk assessment, clinical follow-up, health education, self-care promotion, and adherence counseling. For example, the Heart Outcomes Prevention Evaluation 4 (HOPE-4) trial, a cluster-randomized community-based study, demonstrated that a CHWs-led intervention achieved systolic blood pressure control (<140 mmHg) in 69% of participants compared to 30% in the standard care group (*p* < 0.0001) (9). Such community-centered approaches are cost-effective for managing chronic diseases and offer a pathway to overcoming access barriers and promoting health equity. Community empowerment also has the potential to enhance health and well-being through strategies such as home visits, integration with medical consultations, and improved access to comprehensive healthcare services (10).

The “Implementation, Integration, and Institutionalization of a Community-Based Care Program to Reduce Cardiovascular Risk in Santander” (RE-HOPE) study will be developed to optimize community-level hypertension management by integrating CHWs into the processes of early detection, follow-up, and blood pressure control. The RE-HOPE model combines detection and referral mechanisms, structured health counseling, ongoing monitoring, and the use of simplified clinical algorithms to facilitate therapeutic decision-making and improve clinical outcomes.

Currently, cardiovascular risk programs at both the regional and national levels have gaps that limit their coverage. First, a high proportion of individuals with hypertension are unaware of their condition and therefore do not access cardiovascular risk programs; on the other hand, although some individuals with hypertension are aware of their disease, participation in institutional activities remains suboptimal. It is estimated that compliance with institutional programs is only around 60% (11). Additionally, among those already enrolled in cardiovascular risk programs, it is necessary to overcome some of the barriers identified by users in order to achieve hypertension control, including access to health services, coverage, education about the disease, and interaction with patients (12). The RE-HOPE model is designed to address these gaps by helping institutional cardiovascular risk programs increase their coverage. This will be achieved by strengthening patient engagement, reinforcing home-based health education, and improving continuity of care through more patient-centered strategies.

The distinguishing feature of the RE-HOPE study is the timely institutionalization of patients into cardiovascular risk management programs through structured coordination between community case detection and primary healthcare providers. We aim to develop an innovative and potentially scalable strategy that, if proven effective, may help close existing gaps in hypertension control in LMICs such as Colombia.

## Methods

### Study design

RE-HOPE will be a sequential mixed methods design study comprising two phases.

In the qualitative phase, we will conduct a study within the interpretive paradigm using a phenomenological design. Focus groups with stakeholders will be conducted using a semi-structured interview guide developed by the research team. Meetings will be held at participants' workplaces, and each session will be audio-recorded, transcribed verbatim, validated, and coded. Eligible professionals will be contacted by telephone and invited to participate voluntarily. Barriers to hypertension control identified during the formative phase, including difficulties in patient identification and challenges in coordination among health system actors affecting the delivery of cardiovascular risk programs, will be incorporated into the intervention design. Accordingly, the intervention seeks to reduce gaps in screening, improve awareness of hypertension, and strengthen healthcare professionals' knowledge of the diagnosis, treatment, and control of hypertension.

The second phase will consist of a quantitative study aimed at assessing the effectiveness of the implemented strategy. A single-arm intervention design will be used to evaluate longitudinal changes in blood pressure through within-participant comparisons of baseline and follow-up measurements.

The RE-HOPE study will be conducted in primary healthcare centers in Santander, Colombia. This manuscript describes the intervention protocol and follow-up procedures implemented by the community health team.

### Implementation general framework

The RE-HOPE study will be conducted within the general framework of the Exploration, Preparation, Implementation, and Sustainment (EPIS) model, which will guide the implementation process and inform the assessment of determinants influencing implementation. The EPIS model is a conceptual implementation framework that facilitates understanding of key contextual factors and their interactions throughout the implementation and sustainment phases. Specifically, EPIS comprises four stages: (1) exploration of the need for change; (2) preparation for intervention adoption; (3) intervention implementation; and (4) intervention sustainment over time (13). Across these four phases, the study will examine factors related to both the inner context (characteristics of healthcare institutions, providers, administrators, and staff) and the outer context (government agencies, communities, pharmacies outside clinics, and insurance companies/providers), as well as patient characteristics that may affect implementation. Table 1 presents the operationalization of the intervention components according to the EPIS framework, including contextual factors, implementation strategies, involved actors, implementation outcomes, and indicators. The RE-HOPE model will adapt a community-based intervention implemented by a working team comprising an operational coordinator, a data manager, community health workers (nursing assistants), community leaders, and healthcare professionals at primary care centers (PCCs). The roles of the team members are described in Table 2. The workload will be organized according to the identification and follow-up of individuals with hypertension in each community to which each nursing assistant is assigned (district or municipality). The individuals with hypertension may be served by one or more PCCs in each district or municipality.

**Table 1:** Implementation of the RE-HOPE approach based on EPIS model.

EPIS phase	Context factor	Implementation strategy	Responsible actors	Implementation outcome	Indicator	Data source/time point
Exploration	Outer context	Engagement with community leaders and health authorities to introduce the RE-HOPE approach	Research team, community leaders, health authorities	Acceptability	Proportion of stakeholders reporting high acceptability of the intervention in the qualitative phase	Structured stakeholder survey; pre-implementation
	Inner context	Assessment of institutional readiness and implementation capacity in primary care centers	Research team, clinic managers	Feasibility	Number of primary care centers meeting readiness criteria	Readiness assessment checklist; pre-implementation
Preparation	Inner context	Training program for CHWs and nursing assistants on standardized BP measurement, counseling, referral procedures, and data entry	Research team, clinical trainers	Feasibility	Percentage of CHWs and nursing assistants completing certification training	Training attendance records; pre-implementation
	Inner context	Definition of operational workload for CHWs, including assignment of households and follow-up responsibilities	Research team, clinic managers	Feasibility	Mean number of households and participants assigned per CHW	Project operational records; pre-implementation
	Bridging factor	Coordination meetings between community teams and primary care cardiovascular risk programs	Research team, clinic managers	Adoption readiness	Number of coordination meetings conducted	Meeting minutes; pre-implementation
	Innovation factor	Development of standardized counseling materials and operational tools for home visits	Research team	Fidelity preparedness	Availability of standardized materials across participating clinics	Project documentation; pre-implementation
Implementation	Innovation factor	Community-based hypertension screening through door-to-door visits and outreach activities	CHWs, community leaders	Adoption	Number of individuals screened for hypertension per CHW	REDCap study database; implementation period
	Bridging factor	Referral pathway connecting community screening with cardiovascular risk programs in primary care	CHWs, primary care providers	Reach	Percentage of screened hypertensive individuals attending primary care appointment	Referral tracking system; implementation period
	Inner context	Delivery of structured home-based counseling sessions on hypertension management and lifestyle modification	CHWs	Fidelity	Proportion of planned home visits completed according to protocol	Visit checklists and REDCap records; implementation period
	Inner context	Monitoring and supervision of CHWs by nursing supervisors through periodic meetings and field visits	Nursing supervisors	Fidelity	Number of supervision meetings or field visits conducted per CHW	Supervision logs; implementation period
	Inner context	Monitoring of CHW workload during implementation	Nursing supervisors, research team	Feasibility	Average number of home visits conducted per CHW per month	Study database and supervision reports; implementation period
	Inner context	Follow-up visits at months 1, 3, 6, 9, and 12 including BP measurement and counseling	CHWs	Feasibility	Proportion of participants completing scheduled follow-up visits	Study database; implementation period
	Innovation factor	Health counseling sessions addressing medication adherence, physical activity, diet, alcohol reduction, smoking cessation, and stress management	CHWs	Acceptability	Participant satisfaction with counseling sessions	Participant satisfaction survey; implementation period
Sustainment	Inner context	Integration of RE-HOPE activities into existing cardiovascular risk management programs	Health authorities, clinic administrators	Sustainability	Inclusion of RE-HOPE protocol within institutional care pathways	Institutional documents; post-implementation
	Outer context	Evaluation of barriers and facilitators for long-term implementation and scale-up	Research team, health system stakeholders	Institutionalization	Identification of facilitators and barriers for future scale-up	Semi-structured interviews; post-implementation

BP, Blood pressure; CWHs, Community Health Workers.

**Table 2:** Role of team members.

Members	Function	Activities
Nurse, Operational Coordinator	Management in the territory	Coordinate the deployment of nursing assistants and community leaders.
		Liaise with PCCs and maintain communication.
		Conduct training for nursing assistants, community leaders, and health workers at PCCs.
		Supervise the activities of nursing assistants and the health counselling.
		Supervise data collection.
Community Health Workers-Nursing assistant	Monitoring of hypertensive individuals. Data collection	Conduct home visits, with standardized blood pressure measurements.
	Health counselling	Offer health counselling according to individual educational needs.
		Fill out follow-up forms.
		Report any new developments or alarming situation.
		Provide reminders.
Data manager	Systematization of data	Supervise data collection.
		Centralize data
		Generate reports.
Community leaders.	Liaise with community.	Facilitate contact between nursing assistants and people in the community.
		Carry out logistical tasks to enable home visits to take place.
Health workers in PCC	Institutionalize the person with hypertension	Schedule medical appointments.
		Provide reminders.
		Enroll individuals in institutional cardiovascular risk programs.
		Prescribe antihypertensive medication.

PCC, Primary Care Center

### Population and sample

Adults aged ≥18 years be selected through convenience sampling from the community based on the following criteria: a) newly diagnosed hypertension (defined as blood pressure ≥140/90 mmHg); b) uncontrolled hypertension (defined as blood pressure ≥140 mmHg in individuals with a previous diagnosis of hypertension or who are currently on antihypertensive treatment); and/or c) known hypertensive individuals not regularly attending cardiovascular risk management programs at primary care centers. Exclusion criteria include established cardiovascular disease (e.g., coronary artery disease, myocardial infarction, heart failure, arrhythmias, cardiomyopathies, peripheral arterial disease, stroke or transient ischemic attack, diabetes mellitus with macrovascular complications, or history of invasive cardiovascular procedures), chronic kidney disease requiring dialysis, intolerance to antihypertensive medications, and resistant or secondary hypertension. The exclusion of these people was based on the fact that these pathologies require individualized and specialized management at high levels of care, which exceeds the capabilities of a community-based strategy.

#### Intervention

**Figure 1:**
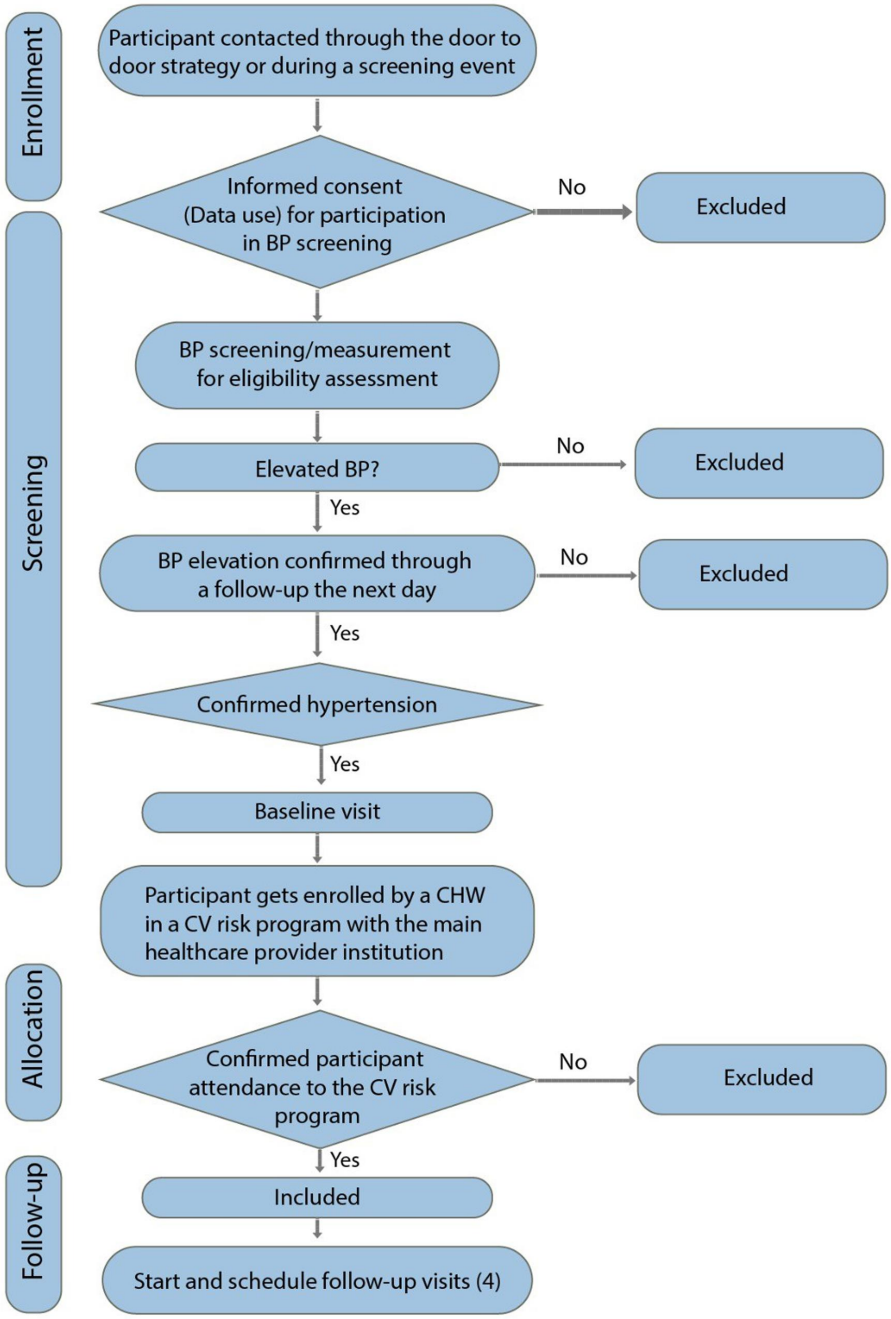
RE-HOPE study flowchart. Trained CHWs, supported by recognized community leaders, will conduct a door-to-door active community-based case finding using a standardized screening protocol. If hypertension is detected, a follow-up measurement will be scheduled within a week. Participants with hypertension will be referred to their assigned primary care provider based on their health insurance affiliation, using a standardized referral form. Once enrolled in a cardiovascular risk management program, home visits will be scheduled as part of the community-based intervention. BP, blood pressure; CHW, community health workers; CV, cardiovascular.

The intervention will include the following components:

Identification of individuals with new or uncontrolled hypertension: RE-HOPE will strengthen the primary network through cardiovascular risk programs, which will promote its integration and institutionalization, a key step for the success of its implementation. In this framework, trained CHWs, supported by community leaders, will conduct active case finding in the community using a standardized screening protocol (Figure 1). Community leaders will facilitate engagement between CHWs and potential participants, organizing meetings, follow-up routes, and actions to increase access and coverage of healthcare services. Screening will occur door-to-door, during community outreach events, and at primary care facilities. After consent, individuals will undergo initial blood pressure measurement. If elevated blood pressure or another inclusion criterion is detected, a follow-up measurement will be scheduled within a week. Individuals with normal blood pressure will receive health education on healthy lifestyles and age-appropriate blood pressure monitoring recommendations (2) Those meeting hypertension criteria will be invited to participate, and informed consent will be obtained. Baseline sociodemographic and clinical data will also be collected.

**Figure 2:**
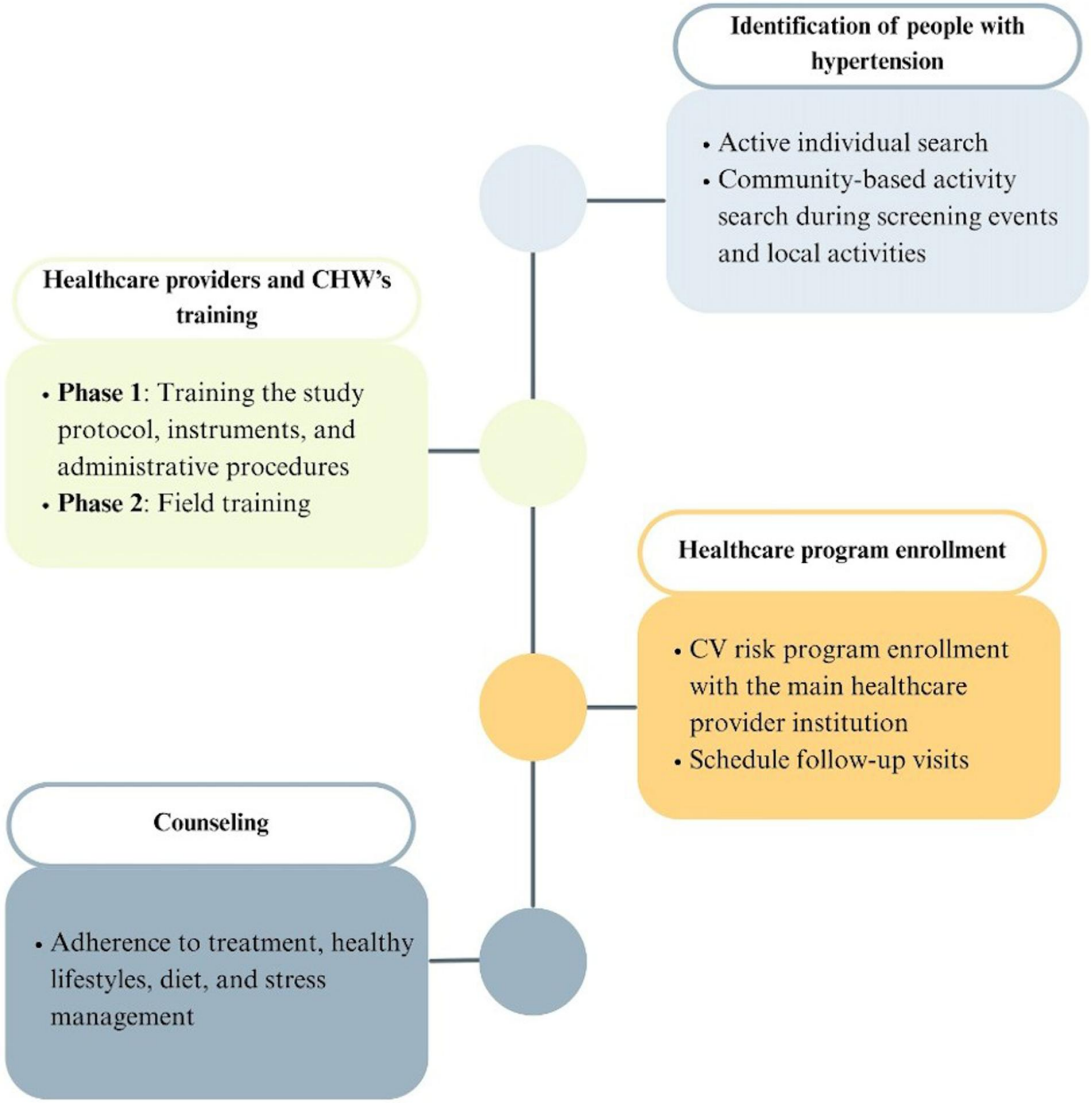
Components of the RE-HOPE intervention strategy. The strategy consists of four sequential components: a) active identification of individuals with hypertension through individual and community-based screening; b) referral to primary healthcare services with a cardiovascular risk management approach; c) training of the implementation team in the study protocol, tools, and procedures; and d) health counseling focused on treatment adherence, healthy lifestyles, and stress management. CHW, community health workers; CV, cardiovascular.

Referral to primary care providers: Participants with hypertension will be referred to their assigned primary care provider based on their health insurance affiliation, using a standardized referral form. A centralized team will submit participant lists via an online platform (e.g., OneDrive) to schedule medical appointments and ensure program enrollment. Participants and/or their families will be contacted by phone with appointment details. If a participant does not attend, they will be followed up to identify barriers and facilitate access to care. If a participant does not agree to assist, they will be excluded. Once enrolled in a cardiovascular risk management program, home visits will be scheduled as part of the community-based intervention (Figure 2).

**Figure 3:**
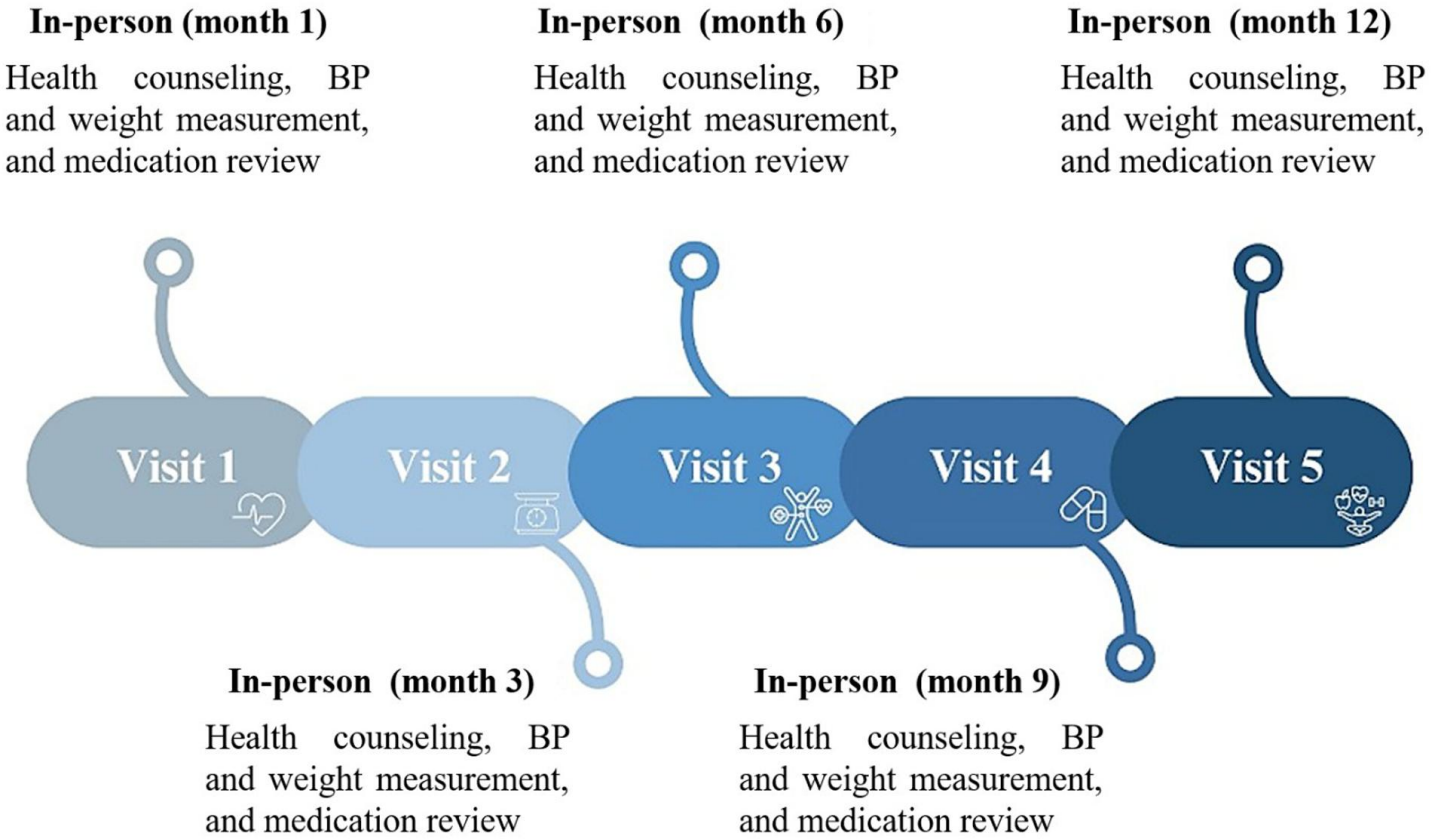
Follow-up visit schedule. The intervention includes five scheduled follow-up visits over 12 months. At each visit, participants will receive health counseling, blood pressure measurement, weight monitoring, and a review of their pharmacological treatment. BP, blood pressure.

Home-based health counseling: CHWs will conduct home visits at months 1, 3, 6, 9, and 12 (Figure 3). Each visit will include an assessment of health education needs and a brief (approximately 20 min) structured counseling session on key hypertension-related topics. Counseling materials will be developed and validated by healthcare professionals with expertise in health sciences. CHWs will receive training before delivering the sessions for a Nurse whit high level education. The counseling sessions will cover the following topics:

1. Disease awareness: CHWs will explain the diagnosis of hypertension in clear, accessible language, emphasizing its importance and its relationship to cardiovascular disease.
2. Medication adherence: Participants will be encouraged to take their medications daily, supported by tools such as pillboxes, medication cards, or reminders tailored to their preferences.
3. Physical activity promotion: Participants will be advised to engage in at least 150 min of moderate-intensity or 75 min of vigorous-intensity physical activity per week, including isometric strength training (14).
4. Reduced sodium intake: Participants will be counseled to limit their intake of processed and packaged foods while increasing their consumption of fruits, vegetables, and home-prepared meals.
5. Reduced alcohol consumption: Limit alcohol intake to one drink per day for women and two drinks per day for men.
6. Tobacco and vape cessation: CHWs will encourage participants to quit tobacco and vaping products.
7. Stress and emotion management: CHWs will help participants identify stressors at home and suggest simple, comforting activities to manage stress.

Healthcare provider training: The research team will train CHWs in standardized blood pressure measurement techniques, strategies to enhance medication adherence, the use of data collection tools, health counseling, and assertive community engagement. Ongoing training sessions will also be held for healthcare professionals and technicians at primary care centers receiving study participants. Topics will include hypertension detection and management based on World Health Organization (WHO)-recommended fixed-dose combination therapy algorithms (15). The training is provided by health professionals who serve as operational coordinator and others health professionals.

### Follow-up

CHWs will arrange appointments for home follow-up visits (months 1, 3, 6, 9, and 12), and blood pressure will be measured according to the standardized protocol. Additional data will be collected on anthropometric variables, physical activity habits, tobacco and alcohol use. The follow-up framework aligns with the guidelines of the International Society of Hypertension for hypertension management and the WHO guidelines (2, 15). Barriers to accessing antihypertensive medications and statins within the healthcare system will also be evaluated. If participants are found to have uncontrolled hypertension, they will be referred to their corresponding primary care center for medical evaluation and adjustment of antihypertensive therapy. In cases of critically elevated blood pressure (>180/100 mmHg), with or without signs of target organ damage, emergency care will be recommended.

### Outcomes and measurements

The primary outcome is the change in systolic blood pressure levels from baseline to 12 months.

Secondary outcomes include:

− Systolic and diastolic blood pressure levels from baseline to 12 months.
− Proportion of participants achieving systolic blood pressure control (<140 mmHg) from baseline to 12 months.
− Changes in the prevalence of healthy behaviors (including reduced tobacco use and alcohol consumption, and regular physical activity).
− Proportion of non-institutionalized individuals with hypertension (i.e., those not enrolled in cardiovascular risk management programs).
− Change in cardiovascular risk score using the WHO risk prediction tools (16).
− Identification of barriers and facilitators related to cardiovascular risk management.
− Change in CHWs' cognitive and attitudinal competencies, assessed through knowledge, attitudes, and practices questionnaires administered to primary care health personnel.

### Data collection

CHWs will collect baseline data, health counseling activities, and follow-up visit information using digitized forms via the REDCap® system. Data collected will include:

- Sociodemographic variables: age, sex, and health insurance affiliation.
- Lifestyle behaviors: tobacco/vape use, alcohol consumption, and weekly physical activity.
- Clinical measures: systolic and diastolic blood pressure.
- Anthropometric measurements: weight, height, body mass index (BMI), waist circumference, hip circumference, and waist-to-hip ratio.
- Cardiovascular risk scores: calculated using WHO tools.
- Laboratory results will be obtained from the clinical laboratories of the assigned primary care providers, as recorded in individual service provision records.

### Blood pressure measurement

Blood pressure will be assessed using a strictly standardized protocol. Participants will be instructed to avoid caffeine, smoking, and physical activity for at least 30 min before the assessment. The appropriate cuff size will be selected according to mid-arm circumference, and blood pressure readings will be recorded using a calibrated device in accordance with the manufacturer's specifications. Blood pressure will be measured with the participant seated comfortably after a rest period of at least 5 min in a quiet environment. During the measurement period, participants will remain seated with their back supported, feet flat on the floor, and legs uncrossed. The arm used for measurement will be uncovered and supported at heart level. Three consecutive measurements will be obtained at 1- to 2-minute intervals using validated automated upper-arm devices. For analysis, the average of the last two readings will be used to improve accuracy and reduce short-term variability. To minimize misclassification related to the white-coat effect, out-of-office blood pressure monitoring will be recommended when clinically indicated. All procedures will be conducted in accordance with the recommendations of the International Society of Hypertension (ISH) guidelines, ensuring methodological rigor and reproducibility (2).

### Sample size

Based on the findings of a meta-analysis by Mills et al. (8), the primary efficacy outcome sample size calculation assumed a 7-mmHg reduction in SBP (SD 50) after 12 months of follow-up, and a correlation of 0.3 between baseline and follow-up measurements. Using 90% statistical power and a two-sided significance level of 5%, the required initial sample size was estimated at 1,912 participants, increasing to a final sample size of 2,294 after allowing for approximately 20% lost, with participants recruited proportionally across provinces.

### Analysis plan

#### Qualitative analysis

A deductive analysis will be conducted using Nvivo software, version 1.6.1., through open coding of focus group narratives. Iterative reading will enable the identification and refinement of core categories (facilitators and barriers) and emerging subcategories until thematic saturation is achieved. During the interview process, credibility will be ensured using follow-up questions predefined in the interview guide. Subsequently, the findings will be discussed with two participants to ensure confirmability.

#### Clinical effectiveness outcomes

Descriptive analyses will summarize continuous variables using means or medians, along with standard deviations or interquartile ranges, depending on the distribution of the data. Absolute and relative frequencies represent categorical variables. For the primary and secondary outcomes, the analysis will produce estimates using linear mixed models of the continuous outcomes (change in systolic blood pressure, systolic and diastolic blood pressure), and generalized linear mixed models for the categorical outcomes (proportion of hypertension control, proportion of modification of life styles, proportion of participants in low, moderate, high and extremely high cardiovascular risk), estimating mean and 95% confidence intervals at baseline and 3, 6, 9, and 12 months of follow-up. The models will include random intercepts accounting for within-subject correlation over time, and the fixed effects will include sex, region, age and anthropometric measures. We will impute missing covariate data using the predictive mean matching algorithm implemented in the mice package (version 3.16) in R statistical software (version 4.3). We will not impute missing outcome because these are expected to be approximately missing at random, primarily due to participants moving out of their municipality of residence. Accordingly, the mixed-effects model will be fitted using the observed data. Finally, we will perform a sensitivity analysis comparing the primary and secondary outcomes between participants who are aware of their condition and receiving treatment and those who are unaware of their condition. The mixed-effects models will be fitted using the lme4 (version 1.1-35.1) and mgcv (version 1.9-1) packages in R.

### Implementation outcomes

Implementation fidelity will be assessed to determine whether home visits and planned CHW-led activities were carried out in accordance with the protocol. Coverage will be measured as the proportion of the target population reached, and acceptability will be evaluated from the perspectives of participants, CHWs, and healthcare personnel. Operational feasibility will be examined by identifying logistical, administrative, and contextual barriers and facilitators. Finally, the medium- and long-term sustainability of the strategy, as well as its scalability to other settings, will be explored to support its integration into the primary healthcare system (Table 1).

## Expected results

### Primary outcome

The primary efficacy outcome will be the change in systolic blood pressure from baseline to the 12-month follow-up visit. Based on findings from previous community-based interventions, we anticipate at least a 7 mmHg reduction in systolic blood pressure after 12 months of follow-up (8, 9).

### Secondary outcomes

After 12 months, the intervention will be assessed for its impact on blood pressure control (systolic blood pressure <140 mmHg) with a target of control around 60% of participants and the adoption of healthy behaviors, including increased physical activity and reduced tobacco and alcohol use compared to baseline. Improvements in participants' understanding of hypertension, its treatment, and the importance of regular medical follow-up may be observed. Additionally, the strategy will be evaluated for its potential to reduce the incidence of fatal and non-fatal myocardial infarction and stroke. The implementation of CHWs may contribute to improve participants' perceptions of support, trust, and accessibility to primary healthcare.

### Process and implementation outcomes

The RE-HOPE strategy is expected to serve as a replicable and sustainable model for improving hypertension control in resource-limited settings, promoting more equitable and community-centered healthcare delivery.

## Discussion

The RE-HOPE study is proposed as an innovative intervention in Colombia and potentially applicable to other low- and middle-income countries in Latin America. The identification and referral of individuals with hypertension to their primary healthcare providers is a distinguishing feature of this strategy. Community-based strategies involving CHWs have proven effective in improving healthcare access and treatment adherence by reducing geographic, economic, and cultural barriers, ultimately decreasing cardiovascular risk in vulnerable populations (17). RE-HOPE integrates community-based screening, health counseling, and home-based follow-up to improve hypertension control. In individuals with hypertension, community-based models that incorporate education and health counseling have demonstrated potential to enhance prevention and health promotion strategies by fostering trust, increasing treatment adherence, and improving clinical outcomes (10, 17). A recent example is the Hypertension Treatment in Nigeria program, conducted across 60 primary care centers, which involved CHWs in quarterly follow-ups of individuals with hypertension. After an average follow-up of 3.8 years, the intervention resulted in a significant increase in medication adherence (from 71.5% to 85%; *p* < 0.001) and hypertension control (from 78.4% to 84.4%; *p* = 0.009) (18). The RE-HOPE model will incorporate evidence-based health counseling as a tool to promote lifestyle changes and reduce modifiable risk factors, such as physical inactivity and sodium intake. The inclusion of structured education during home visits is expected to strengthen continuity of care and enhance long-term control of hypertension.

Evaluating the implementation capacity of the RE-HOPE strategy is essential to ensure its effectiveness, sustainability, and adaptability to the regional context. This evaluation will assess whether the components of the strategy—CHWs training, patient engagement, and resource availability—can be executed as planned. Additionally, RE-HOPE aims to generate evidence on the barriers and facilitators of hypertension management to inform timely adjustments and optimize outcomes. In resource-limited settings, RE-HOPE could serve as a primary care strengthening strategy, reduce health inequities, and lower the burden of cardiovascular disease. There is growing evidence that combining active screening, education, and continuous follow-up reduces the burden of hypertension in underserved communities, constituting a scalable approach suitable for other contexts (10). For instance, the Control of Blood Pressure and Risk Attenuation (COBRA-BPS) trial conducted in Bangladesh, Pakistan, and Sri Lanka assessed a multicomponent CHWs-led intervention in rural communities. CHWs played a key role in delivering hypertension education, promoting treatment adherence, and performing regular community-based blood pressure monitoring, thereby facilitating early detection and continuous follow-up. The intervention was cost-effective, with acceptability curves ranging from 79.3% to 99.8%, and incremental cost-effectiveness ratios below the countries' gross domestic product thresholds (19). Such models are effective in increasing awareness and control of hypertension, particularly among individuals with low educational attainment and limited access to healthcare, characteristics common among the RE-HOPE target population.

In the context of Colombia, where healthcare access is unequal, especially in rural areas, strategies like RE-HOPE are crucial for informing evidence-based public policy decisions. Multicomponent interventions led by CHWs are not only clinically effective but also cost-effective (20). The implementation of RE-HOPE represents a strategic opportunity to strengthen primary care, reduce the burden of cardiovascular disease, and promote a more equitable, preventive, and community-centered health system. Its rigorous evaluation will generate locally applicable knowledge that can be scaled to other regions in Colombia and Latin America, contributing to the development of more resilient and efficient health systems.

Despite its strengths and potential contributions to hypertension monitoring and control, this study will have some limitations. The single-arm design may limit our ability to attribute observed changes exclusively to the intervention because no comparator group will be included. In addition, methodological aspects such as convenience sampling may introduce selection bias and reduce the representativeness of the sample relative to the broader population of individuals with hypertension in Santander. It is important to note, however, that the strategy is specifically intended to identify and recruit individuals who do not usually access healthcare services through screening in primary care facilities. Furthermore, the exclusion of individuals with established cardiovascular disease and resistant hypertension may limit the generalizability of the findings to populations with more severe cardiovascular conditions. Nevertheless, the intervention was specifically designed for large-scale implementation in community settings within the primary care system rather than at higher levels of care. Accordingly, the findings should be interpreted with caution when extrapolating them to the overall population of individuals with hypertension.

## References

[1] Mills KT Stefanescu A He J. The global epidemiology of hypertension. Nature reviews nephrology. Nat Res. (2020) 16:223–37. 10.1038/s41581-019-0244-2 PMC7998524 32024986

[2] Unger T Borghi C Charchar F Khan NA Poulter NR Prabhakaran D 2020 International society of hypertension global hypertension practice guidelines. J Hypertens. (2020) 38:982–1004. 10.1097/HJH.0000000000002453 32371787

[3] Roth GA Mensah GA Johnson CO Addolorato G Ammirati E Baddour LM Global burden of cardiovascular diseases and risk factors, 1990–2019: update from the GBD 2019 study. J Am Coll Cardiol. (2020) 76:2982–3021. 10.1016/j.jacc.2020.11.010 33309175 PMC7755038

[4] Joseph P Lanas F Roth G Lopez-Jaramillo P Lonn E Miller V Cardiovascular disease in the Americas: the epidemiology of cardiovascular disease and its risk factors. Lancet Reg Health Am. (2025) 42:100960. 10.1016/j.lana.2024.100960 40034110 PMC11873637

[5] Lopez-Jaramillo P Lopez-Lopez JP Otero J Alarcon-Ariza N Mogollon-Zehr M Camacho PA May measurement month 2019: an analysis of blood pressure screening results from Colombia. Eur Heart J Suppl. (2021) 23(Supplement_B):B46–8. 10.1093/eurheartj/suab039 34248430 PMC8263072

[6] Irazola V Prado C Rosende A Flood D Tsuyuki R Ojeda CN Expanding team-based care for hypertension and cardiovascular risk management with HEARTS in the Americas. Rev Panam Salud Publica. (2025) 49:e43. 10.26633/RPSP.2025.43 40357407 PMC12065422

[7] Schwalm JD Joseph P Leong D Lopez-Lopez JP Onuma O Bhatt P Cardiovascular disease in the Americas: optimizing primary and secondary prevention of cardiovascular disease series: cardiovascular disease in the Americas. Lancet Reg Health Am. (2025) 42:100964. 10.1016/j.lana.2024.100964 40034111 PMC11873640

[8] Mills KT Obst KM Shen W Molina S Zhang HJ He H Comparative effectiveness of implementation strategies for blood pressure control in hypertensive patients: a systematic review and meta-analysis. Ann Int Medi. (2018) 168:110–20. 10.7326/M17-1805 PMC5788021 29277852

[9] Schwalm JD McCready T Lopez-Jaramillo P Yusoff K Attaran A Lamelas P A community-based comprehensive intervention to reduce cardiovascular risk in hypertension (HOPE 4): a cluster-randomised controlled trial. Lancet. (2019) 394(10205):1231–42. 10.1016/S0140-6736(19)31949-X 31488369

[10] Viswanathan M Kraschnewski JL Nishikawa B Morgan LC Honeycutt AA Thieda P Outcomes and costs of community health worker interventions: a systematic review. Med Care. (2010) 48(9):792–808. 10.1097/MLR.0b013e3181e35b51 20706166

[11] Lamelas P Diaz R Orlandini A Avezum A Oliveira G Mattos A Prevalence, awareness, treatment and control of hypertension in rural and urban communities in Latin American countries. J Hypertens. (2019) 37(9):1813–21. 10.1097/HJH.0000000000002108 30964825

[12] Legido-Quigley H Lopez PAC Balabanova D Perel P Lopez-Jaramillo P Nieuwlaat R Patients’ knowledge, attitudes, behaviour and health care experiences on the prevention, detection, management and control of hypertension in Colombia: a qualitative study. PLoS One. (2015) 10(4):e0122112. 10.1371/journal.pone.0122112 25909595 PMC4409332

[13] Moullin JC Dickson KS Stadnick NA Rabin B Aarons GA. Systematic review of the exploration, preparation, implementation, sustainment (EPIS) framework. Implement Sci. (2019) 14:1. 10.1186/s13012-018-0842-6 30611302 PMC6321673

[14] Cohen DD Aroca-Martinez G Carreño-Robayo J Castañeda-Hernández A Herazo-Beltran Y Camacho PA Reductions in systolic blood pressure achieved by hypertensives with three isometric training sessions per week are maintained with a single session per week. J Clin Hypertens. (2023) 25(4):380–7. 10.1111/jch.14621 PMC10085809 36965163

[15] Al-Makki A DiPette D Whelton PK Murad MH Mustafa RA Acharya S Hypertension pharmacological treatment in adults: a world health organization guideline executive summary. Hypertension. (2022) 79(1):293–301. 10.1161/HYPERTENSIONAHA.121.18192 34775787 PMC8654104

[16] Lopez-Lopez J Garcia-Pena A Martinez-Bello D Gonzalez A Perez-Mayorga M Munoz-Velandia O External validation and comparison of six cardiovascular risk prediction models in the prospective urban rural epidemiology (PURE)-Colombia study. Eur J Prev Cardiol. (2025) 32:573–4. 10.1093/eurjpc/zwae286 39041366 PMC12066169

[17] Kim K Choi JS Choi E Nieman CL Joo JH Lin FR Effects of community-based health worker interventions to improve chronic disease management and care among vulnerable populations: a systematic review. Am J Public Health. (2016) 106:e3–28. 10.2105/AJPH.2015.302987 26890177 PMC4785041

[18] Shedul GL Ripiye N Jamro EL Orji IA Shedul GJ Ugwuneji EN Supportive supervision visits in a large community hypertension programme in Nigeria: implementation methods and outcomes. BMJ Open Qual. (2025) 14(1):e003163. 10.1136/bmjoq-2024-003163 40127954 PMC11934367

[19] Finkelstein EA Krishnan A Naheed A Jehan I de Silva HA Gandhi M Budget impact and cost-effectiveness analyses of the COBRA-BPS multicomponent hypertension management programme in rural communities in Bangladesh, Pakistan, and Sri Lanka. Lancet Glob Health. (2021) 9(5):e660–7. 10.1016/S2214-109X(21)00033-4 33751956 PMC8050199

[20] Zhang Y Yin L Mills K Chen J He J Palacios A Cost-effectiveness of a multicomponent intervention for hypertension control in low-income settings in Argentina. JAMA Netw Open. (2021) 4(9):E2122559. 10.1001/jamanetworkopen.2021.22559 34519769 PMC8441594

